# Diabetic Ankle Fractures: A Review of the Literature and an Introduction to the Adelaide Fracture in the Diabetic Ankle Algorithm and Score

**DOI:** 10.1155/2014/153146

**Published:** 2014-01-05

**Authors:** Joshua Yee, Anand Pillai, Linda Ferris

**Affiliations:** ^1^Department of Orthopaedics and Trauma, The Queen Elizabeth Hospital, 28 Woodville Road, Woodville West, SA5011, Australia; ^2^South Manchester University Hospital, South Moor Road, Wythenshaw, Manchester, UK; ^3^The Queen Elizabeth Hospital, Woodville West, SA5011, Australia

## Abstract

Diabetics who have acquired an ankle fracture may be easily missed given their atypical presentation. As such, it is not infrequently seen that these patients are either initially misdiagnosed or ineffectively managed resulting in unnecessary hospital length of stay and procedures. Multiple review articles and retrospective studies have been previously published in the literature, but complete guidelines to assist in accurate diagnosis and cost-effective management for this complex problem do not currently exist. Through a critical analysis of the current literature, a proposed diagnostic and management algorithm and scoring system that can be used to quantify risks in the surgical management are presented for consideration.

## 1. Introduction

Both diabetes and ankle fractures are increasing in incidence worldwide. In 2003, it was estimated that 194 million people in the world have been diagnosed with diabetes and this is predicted to exponentially increase to over 333 million by 2025 [1]. Ankle fractures are amongst the most common injuries encountered by orthopaedic surgeons, with its incidence on the rise in parallel with general life expectancy [2, 3]. Extrapolated from this data, the incidence of diabetic ankle fractures will inevitably increase. There is, however, a lack in a standardized diagnostic and treatment guidelines for diabetic ankle fractures. Additionally and not uncommonly, diabetic ankle fractures have been misdiagnosed resulting in delayed management. For these reasons, we propose a diagnostic and management algorithm that incorporates a quantitative scoring system in hope to achieve a practical approach to this complex and challenging problem.

## 2. Materials and Methods

Publications were identified by conducting a comprehensive keyword search of Medline, EMBASE, and CINAHL databases between the months of September 2010 to March 2011 by the primary author (Joshua Yee). Search terms included “diabetes,” “ankle,” and “fracture.” Available abstracts of all articles (i.e., no date restriction to search results) published in the English language in the above databases searched within the above timeframe where included for initial review to determine suitability. Inclusion criteria for full-text review were that articles must be relevant to diabetic ankle fracture diagnosis and management. Manual search of the all the references in the full-text publications was also completed to further identify additional publications for potential inclusion. All included full-text publications for review were further defined as either a notable or supportive. A publication was defined to be notable if the study design (i.e., cohort, retrospective, case control, and case series) and level of prognostic strength (i.e., level IV or higher) were met. Notable publications were subjected to further critical analysis. Exclusion criteria were the following: publications not published in English; all conferences, lectures, review articles, and publications that were neither published nor published in the above databases. Duplicate results that occurred in different databases were truncated to a single result.

## 3. Results

**Figure 1:**
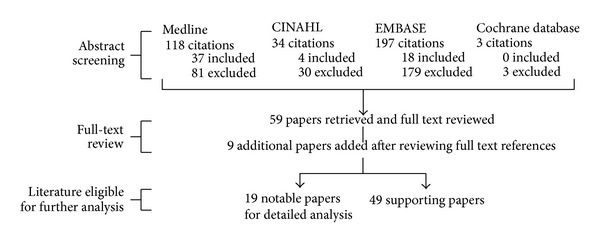
Literature review results in detail.

**Figure 2:**
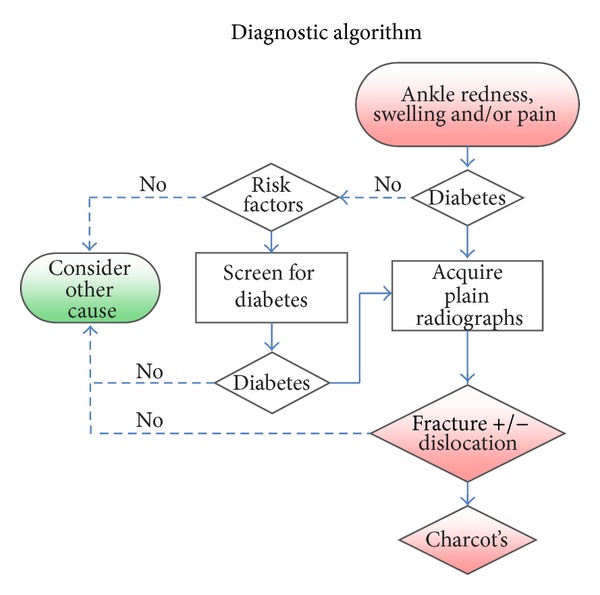
AFDA diagnostic algorithm.

**Figure 3:**
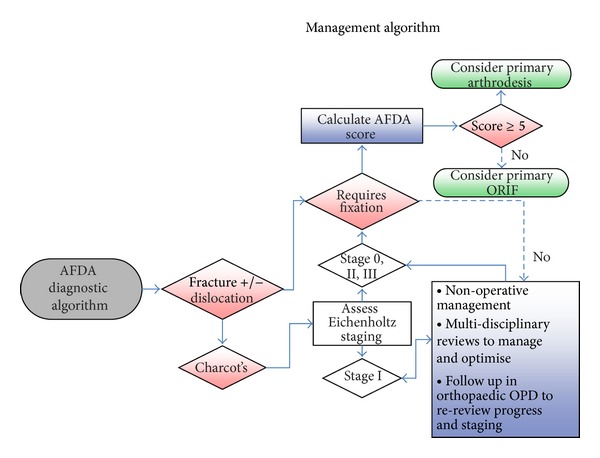
AFDA management algorithm.

A total of 352 abstracts were initially screened. From these abstracts, 59 articles met our inclusion criteria for further full text review. 293 articles were excluded. Nine additional articles were found after reviewing all the full-text references (Figure 1). Nineteen notable papers were identified for further critical analysis (Tables 1 and 2). From these results, we propose the Adelaide Fracture in the Diabetic Ankle (AFDA) algorithm and score (Figures 2 and 3, Table 3).

## 4. Discussion

The AFDA algorithm consists of two parts: a diagnostic and management part (Figures 2 and 3), having its own respective goals. The diagnostic part is targeted for primary care physicians and nurse practitioners with a goal to accurately and timely provide a diagnosis to any presentation of ankle redness, swelling, and/or pain; and if such a presentation is encountered, the algorithm progresses to assess or screen for the presence and risks for diabetes.

Risk factors for diabetes include previous history of impaired glucose intolerance; high-risk ethnic groups (i.e., Asian, African, and Hispanic); positive family history or gestational diabetes; age greater than or equal to 45 with either a body mass index (BMI) greater or equal to 25 or presence of hypertension; and established cardiovascular risk factors or disease. If risk factors are present, screening for diabetes is suggested using either HBA1c (greater or equal to 6.5%), two-hour plasma glucose (greater or equal to 11.1 mmol/L) during an oral glucose tolerance test, or fasting blood glucose (greater or equal to 7.0 mmol/L) [4].

If the presence of diabetes has been excluded, other causes of ankle redness, swelling, and/or pain should be considered, which can include: gout, infection, septic arthritis, deep vein thrombosis (DVT), and haemarthrosis [5, 6]. Furthermore, if there is an absence of systemic signs of infection (i.e., fever, elevated white cell count, or CRP), then infection is unlikely [6].

Since the introduction of the Ottawa ankle rules [7], there has been a significant increase in cost efficiency to plain radiograph usage in assessing acute ankle injuries. However, there has been question of whether the Ottawa ankle rules apply in diabetics [8, 9]. If there is a history of diabetes or newly diagnosed diabetes, we suggest that plain radiographs can be acquired independent of the Ottawa ankle rules. This should be interpreted for presence of a fracture, dislocation, and/or Charcot's arthropathy. Because of the possibility of an insensate lower limb, the patient's history may be difficult to elicit which has occurred first: the fracture or acute Charcot's. However, if any of these are present, the AFDA management algorithm and score can be applied.

The AFDA management algorithm and score will assist the treating specialist in the optimal decision making for managing diabetic ankle fractures. If a fracture requires fixation, the AFDA score (Table 3) can be considered to differentiate between managing it as a primary ORIF or primary rigid fixation/arthrodesis. Ganesh et al. [3] have, in an analysis of a nationwide inpatient database, concluded that there are significant increases in in-hospital length of stay and costs for diabetics with ankle fractures compared with nondiabetics. Having a decision from the outset can potentially decrease these factors and, furthermore, allow the patient to return to their usual activities earlier and save them from unnecessary revision surgery. Rigid fixation/arthrodesis is reserved for patients who are at high risk of amputation or failure if primary ORIF is used. Comparing techniques of rigid fixation/arthrodesis is beyond the scope of this discussion, but options include retrograde intramedullary nailing [10, 11], posterior blade plate [12], external ring fixators [13], and cross-screw techniques [14].

If Charcot's arthropathy is present in conjunction with the diabetic ankle fracture, Eichenholtz's [15] staging should also be considered. As Eichenholtz stage I has traditionally been considered to be the acute fragmentation stage, the current literature recommends that this stage can be managed nonoperatively in the interim, with nonweight bearing and serial total contact casting. Additionally, frequent followup and further patient optimization through multidisciplinary review and management can be considered. Endocrine input for consideration of intravenous bisphosphonate therapy (i.e., Pamindronate) has been shown to inhibit the increased osteoclastic activity that weakens bones during this acute stage [16, 17]. Optimization of diabetic control, nutrition, and vascular status whilst this stage progresses will likely achieve a better outcome. Once Eichenholtz's staging progresses outside of stage I, progression for fixation consideration is suggested. Postoperatively and independent from the surgical fixation technique, the literature suggests that all patients undergo prolonged nonweightbearing for 12 weeks, protected or partial weight bearing for another 8 to 12 weeks, with frequent outpatient followup with plain radiographs initially fortnightly for the first six weeks, then monthly thereafter [18].

The AFDA scoring system (Table 3) is based on assessable patient factors. Two points have been allocated to factors more readily seen in the notable publications to suggest either poor outcomes of standard internal fixation or better outcomes with rigid fixation/arthrodesis. A Semmes Weinstein monofilament 10 g/5.07 at the plantar aspect of either the great toe, first, third, or fifth metatarsal head can be used to assess presence of neuropathy [18]. Presence of vasculopathy can be defined as peripheral oxygen saturations consistently less than 95% or an ABI of less than 0.65 [19]. Obesity can be defined as a BMI of greater than 30. Smoking has not been included as a factor because it increases general surgical risk, independent of fixation technique.

With the use of AFDA algorithm, it can hopefully provide a standardized approach and guide to management for diabetic ankle fractures. The scoring system, in particular, can assist in objectively quantifying risk for both the patient and affected ankle joint so as to allow the treating specialist to have confidence in achieving the best possible outcome. As the score increases, the risks of failure in standard fixation methods increase and consideration of more robust and rigid fixation techniques or fusion should be incorporated. From our review of the literature, we suggest that if the patient scores five or more, rigid fixation/arthrodesis should be considered from the outset. If rigid fixation/arthrodesis fails, despite adequate measures in hope for an optimal outcome, amputation can be considered.

The current evidence of which AFDA is derived from is based primarily on heterogeneous study designs and our center's experience. As such, it will require further validation reviews or trials, incorporating followup and functional outcome scores. Once validated, AFDA can be used as a protocol and research tool for the diagnosis and management of diabetic ankle fractures.

## Figures and Tables

**Table 1:** Notable publications in detail.

Study type	Total number of studies	Publication details
Review articles	3	Wukich and Kline [2]
		Prisk and Wukich [18]
		Myerson and Edwards [6]

Cohort study	1	Guo et al. [20]

Retrospective study	2	SooHoo et al. [21]
		Ganesh et al. [3]

Case control study	6	McCormack and Leith [22]
		Jones et al. [23]
		Flynn et al. [24]
		Blotter et al. [25]
		Kristiansen [26]
		Bibbo et al. [27]

Case series	7	Costigan et al. [28]
		Ayoub [14]
		Holmes and Hill [29]
		Kline et al. [30]
		White et al. [31]
		Schon et al. [32]
		Low and Tan [33]

**Table 2:** Critical analysis of the notable publications.

Study type	Authors, year, and location origin	Prognostic evidence strength	Patient details	Relevant findings
Cohort study	Guo et al. [20] China, 2009	Level II	(i) Retrospectively selected 72 patients (36 preoperatively neglected diabetes, 36 nondiabetic controls) with closed ankle fractures between 01/03 and 09/07 (ii) Recall of patients for prospective review over 12 months (iii) Managed either nonoperatively and operatively (iv) Mean age 54.4	(i) Increased incidence of infection, nonunion, and Charcot's arthropathy (ii) No significant difference in AOFAS and Bray's ankle score between two groups

Retrospective study	SooHoo et al. [21] USA, 2009	Level II	57,183 operatively managed ankle fractures (1,219 were complicated diabetic ankle fractures)	Significant increase in complication rates (wound infection, revision operation, and BKA) in complicated diabetic group
	Ganesh et al. [3] USA, 2005	Level II	160,598 nationwide ankle fractures (9174 diabetic ankle fractures) between 1988 and 2000	Diabetics had significant increase in in-hospital mortality, complications, length of stay, and cost

Case control	McCormack and Leith [22] Canada, 1998	Level III	(i) 52 patients (26 diabetic, 26 control) with closed ankle fractures between 04/90 and 01/99 (ii) Mean age 61 (43–78)	Significant increase in complications in both nonoperative and operative fixation in diabetics
	Jones et al. [23] USA, 2005	Level III	(i) 84 patients (42 diabetic, 42 control) (ii) Mean age 57.1	Significant increase in long-term bracing in diabetics (mean age 53.6, insulin dependant, mean duration of DM 20.3 years, and history of Charcot's)
	Flynn et al. [24] Puerto Rico, 2000	Level III	(i) 98 patients with closed ankle fractures (25 diabetic, 73 nondiabetic) between 01/88 and 31/97 (ii) Mean age 44 (nondiabetic) and 60 (diabetic)	Significant increase in postoperative infection in diabetic group (up to five times), especially with factors: nonoperative management, poor glycaemic control, and neuropathy
	Blotter et al. [25] USA, 1999	Level III	(i) 67 surgically treated ankle fractures in patients (21 diabetic, 46 nondiabetic/control) between 03/85 and 10/96 (ii) 4/21 Webber C, 17/21 Webber B (iii) Mean age 55 (diabetic group) and 53 (nondiabetic/control group)	(i) Significant increase in postoperative complication in diabetic group (43% versus 15%), particularly in the insulin dependent (ii) 2 cases of postoperative Charcot's arthropathy in diabetic population (iii) No diabetic subgroup analysis
	Kristiansen [26] Denmark, 1983	Level III	30 patients (10 diabetic, 20 nondiabetic/control)	Significantly increase in wound infection (60% versus 10%) and hospitalization in diabetics (17 versus 9 days)
	Bibbo et al. [27] USA, 2001	Level III	(i) 59 patients with isolated ankle fractures (13 diabetic, 46 nondiabetic/control) (ii) Mean age 55.1 (diabetic), 40.2 (nondiabetic/control) (iii) Mean followup 46 months (diabetic) and 32 months (nondiabetic/control)	(i) Increased complication rate in diabetics compared to nondiabetics (46% versus 17%) (ii) None required amputation/arthrodesis (iii) No information on presence of diabetic complications

Case series	Costigan et al. [28] USA, 2007	Level IV	(i) 84 diabetic patients with previous ORIF of an ankle fracture over an 8-year period (ii) Mean age 49.5 (iii) Average followup 4.1 years	Significant increase in complications in diabetics with peripheral neuropathy and peripheral vascular disease
	Ayoub [14] Egypt, 2008	Level IV	(i) 17 patients with Charcot arthropathy undergoing tibiotalar arthrodesis (ii) Mean age 61.6 (57–69) (iii) Mean followup 26 months	Fusion rates were higher in patients with O_2_ saturations > 95%, decreased BMI, absence of peripheral neuropathy
	Holmes and Hill [29] USA, 1994	Level IV	(i) Assesses relationship of early diagnosis and treatment in 18 patients with diabetic ankle or foot fracture/dislocations between 05/85 and 05/90 (ii) Mean age 55 (iii) Mean followup 27 months	11/20 had a delay in diagnosis with average time of 1 month between onset of symptoms and diagnosis
	Kline et al. [30] USA, 2009	Level IV	(i) 83 tibial pilon fractures (14 diabetic, 68 nondiabetic) between 01/2005 and 06/2007 (ii) Mean age 47.3 (iii) Length of followup 14.5 months (diabetic) and 12.3 months (nondiabetic)	Significant increase in postoperative complications including infection (71% versus 19%) and nonunion/delayed union (43% versus 16%)
	White et al. [31] USA, 2003	Level IV	(i) 14 open ankle fractures in 13 patients with diabetes between 01/01/1981 and 31/12/2000 (ii) Mean age 54 (29–80) (iii) Mean followup 19 months (iv) 9/13 patients were insulin dependent	9/14 developed wound complications, 6/14 had below knee amputations (4 of these were at least Gustilo Class III open fractures), and 3/14 healed
	Schon et al. [32] USA, 1998	Level IV	28 diabetic neuropathic ankle fractures (15 undisplaced, 13 displaced)	(i) Undisplaced ankle fractures are amenable to nonoperative management without significant complications (ii) Of the 13 displaced ankle fractures, high risk of malunion/nonunion if standard ORIF is used
	Low and Tan [33] Singapore, 1995	Level IV	(i) 93 surgically treated ankle fractures (83 nondiabetic, 10 diabetic) between 01/1992 and 06/1993 (ii) Mean age 67.5 (iii) Mean followup 16.2 months	(i) 5 reported cases of infection (all diabetics) (ii) 2/5 requiring below knee amputation, with at least 1/5 having a history of peripheral neuropathy

**Table 3:** AFDA scoring system.

Two points each	One point each
(i) Peripheral neuropathy/loss of protective sensation [2, 23, 24, 28, 33] (ii) Presence of vasculopathy [2, 14, 28] (iii) Insulin dependence with poor compliance [23–25, 30] (iv) Previous or coinciding history of Charcot's arthropathy in any joint [2, 14, 23]	(i) Diabetic history of greater than 20 years [14, 23] (ii) Presence of nephropathy or retinopathy [6] (iii) Obesity [14, 30] (iv) Poor patient compliance
